# miRNA-103 promotes chondrocyte apoptosis by down-regulation of Sphingosine kinase-1 and ameliorates PI3K/AKT pathway in osteoarthritis

**DOI:** 10.1042/BSR20191255

**Published:** 2019-10-18

**Authors:** Fang Li, Jianhua Yao, Qingqing Hao, Zheping Duan

**Affiliations:** 1 Department of Rheumatism and Immunology, Hebei General Hospital, Shijiazhuang City 050051, Heibei Province, P.R. China; 2 Department of Geratology, The Second Hospital of Hebei Medical University, Shijiazhuang City 050051, Heibei Province, P.R. China

**Keywords:** Apoptosis, miRNA-103, Osteoarthritis, PI3K-AKT, SPHK1

## Abstract

Objectives: The aim of the present study was to determine the effects of miRNA-103 on chondrocyte apoptosis and molecular mechanisms in osteoarthritis (OA) progression. Methods: The cell proliferation, apoptosis, and recovery ability were measured by cell counting kit-8 (CCK-8), flow cytometry, and wound healing assays. The interaction of miRNA-103 and Sphingosine kinase-1 (SPHK1) were determined by using luciferase reporter assay. The expression of mRNA and proteins were measured by qRT-PCR and Western blot. OA rat model was established by surgery stimulation. Results: miRNA-103 expression was significantly increased in the cartilage of OA patients and surgery-induced OA rat models. miRNA-103 transfection into primary rat chondrocytes reduced SPHK1 expression, induced apoptosis, inhibited cell proliferation, and impeded scratch assay wound closure. Moreover, expression of total AKT, and p-AKT were significantly reduced in miRNA-103-overexpressing chondrocytes while SPHK1 up-regulation increased the expression of phosphatidylinsitol-3-kinase (PI3K) and p-AKT, and reversed the proliferation suppression induced by the miRNA-103 mimic. Conclusions: Our studies suggest that miRNA-103 contributes to chondrocyte apoptosis, promoting OA progression by down-regulation of PI3K/AKT pathway through the reduction in SPHK1 activity.

## Introduction

Osteoarthritis (OA) is a broadly ubiquitous joint disorder of arthritis, affecting tens of millions of people worldwide. The cause of OA is mostly the wearing down of the protective cartilage on the ends of the bones [1]. OA mainly affects the joints in the hands, knees, hips, and spine even though it will also damage the other joints. To date, there is no effective therapy to completely prevent the onset and the progression of the OA because of the complexity of the mechanisms. However, the OA symptoms can be well managed after adopting a healthy lifestyle. Moderate active, keeping a healthy BMI, and other treatments may slow progression of the disease and help improve pain and joint function [2]. Chondrocytes are the only cell type existing in healthy cartilage. Therefore, any perturbation of the chondrocytes will afflict the OA development and progression. Indeed, chondrocytes plays a critical role in the degeneration process between anabolism and catabolism of the extracellular matrix (ECM) in the cartilage [3].

MicroRNAs (miRNAs) are a subset of the large family of noncoding RNAs and size of the fragment is approximately 21–25 nucleotides [4,5]. The extensive studies suggested that miRNAs could regulate gene expression at the transcriptional and post-transcriptional levels by direct binding to specific sequences within target mRNAs [6]. Many miRNAs present in a time and space pattern in the physiopathology situation in a tissue-specific or developmental stage-specific expression pattern closely associated with some diseases, such as tumor, inflammation, leukemia, spleen, lungs, lymph nodes, and bone disease. Recent evidence demonstrated that miRNAs may play roles to regulate osteogenic and chondrogenic differentiation and proliferation, eventually influencing the catabolism and anabolism of bone and cartilage, suggesting a role of miRNA in the progression of OA [7]. In OA cartilage, it has been demonstrated that miRNAs play a role in OA progression [8–11]. Miyaki et al. [12] demonstrated that the down-regulation of miR-140 expression in OA cartilage and responsible for IL-1β might contribute to the aberrant gene expression pattern feature of OA. IL-1β induced the miR-146 expression that occurred in low-grade OA cartilage [13]. Abouheif et al. [14] claimed loss of miRNA-103 effectively suppressed rat chondrocyte apoptosis and the down-regulation of Col2a1 induced by IL-1β stimulation. In addition, miRNA-103 can promote the cell apoptosis and inhibit proliferation by directly regulating the SIRT1/p53 signaling pathway in primary human chondrocytes. On the contrary, silencing miRNA-103 by intra-articular injection of lentivirus may attenuate disease progression in a rat model of OA [15]. These above observations have revealed that miRNAs most likely regulate OA progression through all biological events such as membrane homeostasis, cell motility, cell proliferation, differentiation, survival, apoptosis, inflammation, and drug resistance [16–18]. Moreover, miR-103 may play crucial roles in the OA progression. Nevertheless, the functions of miR-103 are still largely unknown and underlying mechanism is less understood.

Sphingosine kinase (SPHK) contains two isoforms: SPHK1 and SPHK2 [19]. Sphingosine can be catalyzed by either SPHK1 or SPHK2 to form sphingosine-1-phosphate [19]. Minashima et al. [20] reported that SPHK1 interaciton with ankylosis protein (ANK)/Myb-binding protein 1a (MYBBP1a) stimulates catabolic events in IL-1β-mediated cartilage degradation, suggesting there is a role of SPHK1 in the progression of OA. Phosphatidylinsitol-3-kinase (PI3K)-protein kinase B (AKT) pathway is an intracellular signal transduction pathway, which has multifactorial functions to promote metabolism, proliferation, cell survival, growth, and angiogenesis in response to extracellular signals. However, the correlation of miR-103 with PI3K-AKT is largely unknown in the OA progression.

In the present study, we aimed to investigate the miRNA-103 expression in OA patients and surgery-induced OA rat models, further we extend to examine the correlation between miRNA-103 and SPHK1, an SPHK ubiquitously expressed, evolutionarily conserved enzyme that catalyzes the phosphorylation of sphingosine to sphingosine 1-phosphate (S1P), and their effects on PI3K/AKT pathway in OA.

## Materials and methods

### Samples

The cartilage of knee joint and caput femoris were obtained from the OA patients of two females and five males with hip OA (55–81 years). The patients (nine females and ten males) with knee OA were aged from 47 to 71 years. Cartilage tissues from patients (11 females and 12 males, 43–72 years) with collum femoris fractures underwent total joint replacement were regarded as controls. OA diagnosis was evaluated based on the medical history and X-rays. The gross, pathologic observation was recorded upon replacement of joint. The control group had healthy joints and cartilage. Our research was approved by the ethics committee in our hospital. Fully informed, written consent was acquired from every participant.

### Surgical stimulation of OA

Ten-week-old Sprague–Dawley male rats (obtained from Hebei Laboratory Animal Center, Hebei, China) were randomly assigned to two groups (*n*=12 in each group). Groups included sham-operated control group (Sham) and OA group. Three rats were randomly killed at 1, 2, 3, and 4 weeks subsequent to the surgery. The OA model was generated via surgery. In short, the ligament cruciatum genu anterius or posterius, the meniscus medialis, and the ligamentum mediale of the posterior rat limbs were eliminated in order to bring about articular deformity, which led to the enhancement of damage to the articular face, as well as deterioration of joint cartilage. The complete knee articulation was obtained to conduct a further evaluation. All animal experiments were approved by the Committee of Animal Experiments of our hospital.

### Separation and cultivation of chondrocytes

Primary chondrocyte was isolated from neonatal Sprague–Dawley rat. Briefly, the rat was killed by CO_2_. The cartilages were eliminated under sterile conditions. Then the attached muscle, connective tissue, as well as perichondrium were removed. The rest of the cartilages were minced and washed three times with PBS. Tissues were digested by trypsin (2.5 g/l) for 15 min following by PBS washing for three times to remove the cartilages. Then the tissue was further digesed with a type II collagenase solution (2 g/l) for 5–10 min at 37°C. The extracted cells were filtered with a nylon mesh sieve following by centrifugation for 8 min at 1000 r/min. The proportion of apoptotic cells were evaluated. The separated chondrocytes were placed in cultivating flasks at 1 × 10^5^ cells/ml. Cells were grown in incubator and the medium was replaced every 2 days.

### Cells and cell culture

Cells were cultured in chondrocyte growth medium (Sigma, U.S.A.) containing Dulbecco’s modified Eagle’s medium (DMEM)/Ham’s F-12 1:1 mixture, with l-glutamine (Thermo Fisher, U.S.A.) supplemented with 10% fetal calf serum (FCS). Cells were maintained at 37°C in a humidified incubator with 5% CO_2_.

### Transient transfection for miR-103 functional assessment

The transfection of miR-103 into the primary chondrocytes was performed with Lipofectamine 3000 (Invitrogen, U.S.A.). Briefly, the cells were cultured in six-well plates at 2.5 × 10^5^ cells/well until the confluence reached 80%. The cells were transfected in DMEM without serum or antibiotics utilizing Lipofectamine 3000, miR-103 inhibitor, miR-103 mimic (Sangon Biotech, China), or controls at 30 nM (Sangon Biotech, China). One day after transfection, the medium was replaced and cells were grown for 48 h before experiments.

### Excessive expression of SPHK1

The plasmid for SPHK1 overexpression was purchased from Addgene. After amplification of the plasmid by transformation into the *Escherichia coli* bacteria, the plasmid was extracted. The plasmid was transfected with Lipofectamine 3000 following the protocol as above.

### Flow cytometry

Apoptosis was measured by flow cytometry. Briefly, 1 × 10^5^ cells the cells were digested in trypsin without EDTA. Then the cells were resuspended in binding buffer with 2 μl of 50 μg/ml propidium iodide (PI) and 2 μl of 20 μg/ml Annexin V-FITC. The reaction was processed for 15 min in the dark. The measurement was performed by a flow cytometer (BD; San Jose, CA, U.S.A.) with 488-nm laser excitation. After cell staining for 1 h, the cell distribution was assessed with Modfit LT software (BD; San Jose, CA, U.S.A.). Cells were taken as apoptotic by PI staining negatively, while annexin V-FITC staining is positive signal. For cell cycle assessment, the transfected cells were fixed in 70% ethanol for overnight at 4°C. Then the cells were washed by PBS and collected by centrifugation. After incubation with RNase (10 μg/ml) for 30 min at 37°C. The cells were stained with PI to exclude the negative signal. Then the cell cycle was evaluated with the BD FACSCalibur, CellQuest (BD, Franklin Lakes, NJ, U.S.A.).

### Cell counting kit-8 assay

The cell counting kit-8 (CCK-8) assay was conducted to examine the cell proliferation. Briefly, the chondrocyte or Hs 819.T cells were transfected with miR-103 mimic or inhibitor, then CCK-8 working solution (10 μl) was added directly into each well for 12 h. Absorbance was detected at 450 nm by a BioTek™ Filters for ELx800™ Absorbance Microplate Reader (Thermo Fisher, U.S.A.).

### Wound healing assay

The wound healing assay was performed to examine the cell recovery capability. Briefly, the cells were seeded in a six-well plate until the confluence reached 100%. A scratch was created in the confluent cells with a 200-microliter sterile pipette tip. After gently rinsing with PBS to remove the debris, the cells were allowed to continue growing for 24 h in the medium without serum. Then the scratch-induced wounds was observed and measured under the bright field microscope. The cell recovery scope was assessed with ImageJ software (NIH, U.S.A.). Results were calculated using the closure percentage from the scratch, original width of the scratch was 100%.

### Luciferase reporter gene assay

The wild-type 3′-untranslated region (UTR) and mutant sequences of SPHK1 were amplified with high fidelity polymerase (Shengong, China) followed by the subcloning into the promoter vector (Promega; Madison, WI, U.S.A.). The constructed plasmids were named as pGL3-SPHK1 3′-UTR-WT and pGL3-SPHK1 3′-UTR-MUT. The cells were seeded in 24-well plate until the confluence reached 70%, then the above plasmid (200 ng) as well as miR-103 mimic were co-transfected using Lipofectamine 3000 (Invitrogen; Carlsbad, CA, U.S.A.). The transfection of pGL3 vector was as the control. For luciferase normalization, co-transfections of the *Renilla* luciferase control reporter vector, pRL-SV40 (Promega; Madison, WI, U.S.A.), were performed in HEK293T. Each experiment was repeated at least three times.

### Real-time RT-PCR

Total RNA was isolated with TRIzol reagent (Invitrogen, Carlsbad, CA, U.S.A.) from cells. One microgram RNA was reverse transcribed to get cDNA with SuperScript IV RT Enzymes (Thermo Fisher Scientific, U.S.A.) and the TaqMan miRNA reverse transcription kit (Applied Biosystems, Foster City, CA, U.S.A.). Quantitative real-time polymerase chain reactions (qPCR) was performed with Maxima SYBR Green in ViiA7 Real-Time PCR System (Life Technologies). The samples were run in triplicate. Transcription of either U6 (for miRNAs) or β-actin (for mRNAs) served as the internal reference. Relative gene expression for genes of interest was calculated using the −ΔΔ*C*_t_ method.

### Western blotting

The protein was extracted with RIPA buffer from the cells. The protein concentration was determined by Pierce™ BCA Protein Assay Kit (Pierce, U.S.A.). Each sample was supplemented with a combination of chondroitinase ABC, keratinase, and keratinase II (Sigma, St. Louis, MO, U.S.A.) without proteinase in order to eliminate GAG prior to electrophoretic isolation. Ten micrograms of protein in each well were separated by 10% via SDS/PAGE on gels following by transfer on to PVDF membranes (Thermo Fisher Scientific, U.S.A.). After blocking with 5% BSA/PBS, primary antibodies were added and incubated overnight (4°C). After PBS washing three times, 10 min each time, the second antibody conjugated to HRP was added and incubated for 1 h. ECL kits (Amersham, Piscataway, NJ, U.S.A.) were used to develop the proteins. β-actin was the internal control. The band intensity was quantified with ImageJ software. Primary antibodies were as follows: SPHK1, cleaved caspase 3, Bcl-2, β-actin, Bax (1:1000 dilution) (Cell Signaling Technology, Danvers, MA, U.S.A.), p-AKT, AKT (1:1000 dilution) (Abcam, Cambridge, MA, U.S.A.), MMP3, MMP13, ADAMTS5, type II collagen, and aggrecan (1:1000 dilution) (Abcam, Cambridge, MA, U.S.A.).

### Statistical analysis

Results were expressed as means ± SD. SPSS13.0 software (SPSS, IL, U.S.A.) was used to analyze the data. Differences between groups were analyzed using Student’s *t* test or One-way analysis of variance (ANOVA) followed by Tukey’s post hoc test. *P*<0.05 was significant difference.

## Results

### miRNA-103 expression was significantly up-regulated in OA cartilage

**Figure 1:**
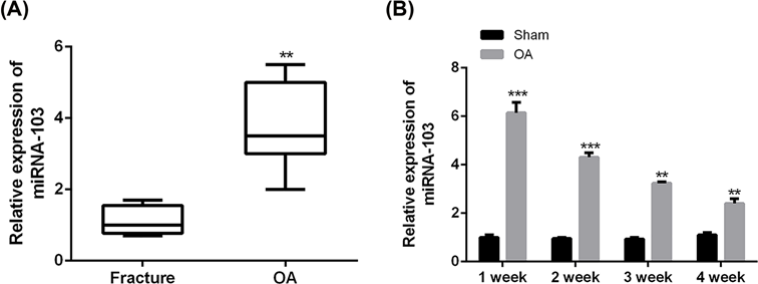
miRNA-103 expression was significantly reduced in OA cartilage (**A**) Relative expression of miRNA-103 levels in cartilage samples of 26 OA patients and 23 OA-free participants was measured by qRT-PCR. (**B**) Relative expression of miRNA-103 levels in cartilage samples of surgery-induced OA rat models was measured by qRT-PCR. ***, *P*<0.001; **, *P*<0.01.

To investigate whether miRNA-103 participated in OA pathogenesis, we first examined miRNA-103 expression in the cartilage of OA patients. The results of Figure 1A confirmed that miRNA-103 expression was significantly increased in OA patients than in OA-free participants (*P*<0.01). Moreover, it was demonstrated that relative expression of miRNA-103 in the cartilage of rat models quickly reached a peak 1 week after surgical induction of OA (Figure 1B).

### miRNA-103 promotes apoptosis in rat primary chondrocytes

**Figure 2:**
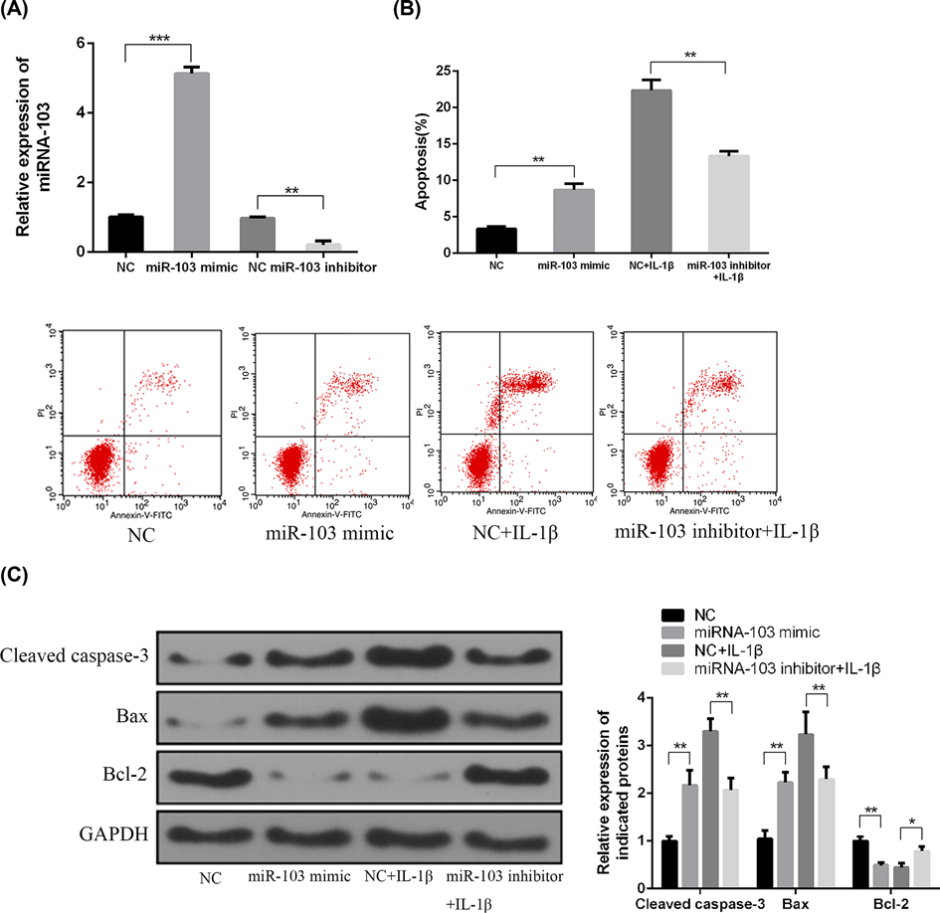
miRNA-103 promoted apoptosis in rat primary chondrocytes (**A**) Relative expression of miRNA-103 in chondrocytes was measured by qRT-PCR. (**B**) Cell apoptosis in chondrocytes was measured by flow cytometry. (**C**) Protein expressions of cleaved caspase-3, Bax, and Bcl-2 were measured by Western blotting. *, *P*<0.05; **, *P*<0.01; ***, *P*<0.001.

To determine the effects of the miRNA-103 on cell apoptosis of chondrocytes, primary chondrocytes were isolated and transfected with an miRNA-103 inhibitor or mimic. The miRNA-103 inhibitor significantly reduced the relative expression of miRNA-103 in chondrocytes (*P*<0.001), while miRNA-103 mimic significantly increased miRNA-103 expression (*P*<0.01; Figure 2A). The apoptosis of chondrocyte was induced by IL-1β (10 ng/ml) to mimic the pathological conditions of OA. The results of flow cytometry showed that miRNA-103 inhibitor significantly suppressed apoptosis induced by IL-1β in primary chondrocytes, whereas apoptotic cells in miRNA-103 mimic-treated group was significantly higher than that in NC group (Figure 2B). In addition, miRNA-103 mimic transfection increased the production of cleaved caspase-3, indicative of apoptosis, and the ratio of Bcl-2/Bax proteins, which play a critical role in cytochrome *c* release from mitochondria and thus initiate apoptosis, while miRNA-103 inhibitor induced opposite effects in primary chondrocytes (Figure 2C).

### miRNA-103 suppressed cell proliferation and migration of rat primary chondrocytes

**Figure 3:**
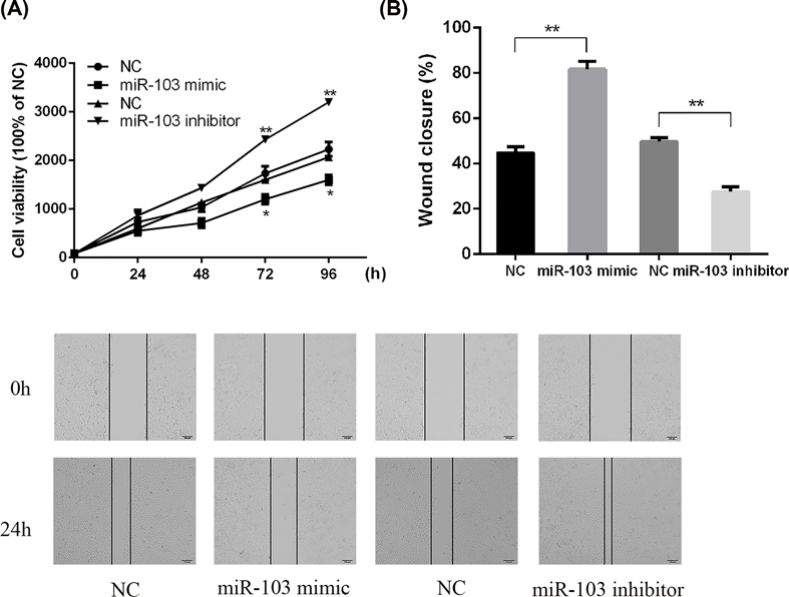
miRNA-103 inhibited the cell proliferation and recovery ability of rat chondrocytes (**A**) Primary rat chondrocytes were transfected with an miRNA-103 mimic or miRNA-103 inhibitor and cell proliferative ability was determined by using CCK-8 assay. (**B**) The cell migration of primary rat chondrocytes was measured by wound healing assay. **, *P*<0.01; *, *P*<0.05.

The effect of miRNA-103 on cell proliferation and migration of primary chondrocytes was examined by using the CCK-8 assay and wound healing assay. As shown in Figure 3A, miRNA-103 mimic resulted in the inhibition of cells proliferation approximately 40% compared with NC after 96-h culture. However, miRNA-103 inhibitor significantly enhanced the cells proliferation in chondrocytes approximately 40% compared with NC. Moreover, the results of wound healing assay revealed that miRNA-103 overexpression significantly reduced the recovery from scratch-induced damage, whereas miRNA-103 down-regulation significantly enhanced the closure of scratch wounds of chondrocytes (Figure 3B). These results suggested that elimination of miRNA-103 might enhance OA cartilage recovery.

### SPHK1 was a direct target of miRNA-103 in rat primary chondrocytes

**Figure 4:**
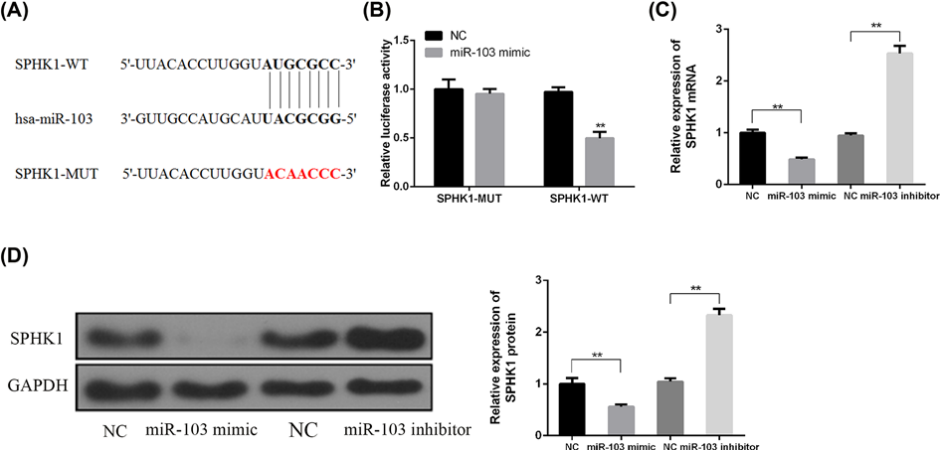
miRNA-103 direct targeted 3′UTR of SPHK1 (**A**) A putative miRNA-103 target site in the 3′-UTR of SPHK1 mRNA was predicted by using bioinformatics analysis. (**B**) Wild-type (SPHK1-WT) or mutant (SPHK1-Mut) luciferase reporter and/or miRNA-103 mimic were co-transfected into HEK293T cells to measure the luciferase activity. (**C**) The cells were treated as indicated, the mRNA level of SPHK1 was measured by RT-PCR. (**D**) The cells were treated as indicated, the protein level of SPHK1 was measured by Western blotting. **, *P*<0.01.

To explore the underlying mechanism of miRNA-103 in OA pathogenesis, the bioinformatics prediction was performed in search of target genes of miRNA-103. According to the prediction results of TargetScan, SPHK1 was found to be a potential target (Figure 4A). Subsequently, the miRNA-103-binding region was mutated in the SPHK1 3′-UTR to verify the interaction between miRNA-103 and the SPHK1 3′-UTR. The wild-type SPHK1 luciferase vector (SPHK1-WT) and mutant type SPHK1 luciferase vector (SPHK1-MUT) were co-transfected with miRNA-103 mimic into HEK293T cells. As shown in Figure 4B, the relative luciferase activity was significantly reduced by miRNA-103 mimics in HEK293T cells transfected with the SPHK1 (WT) vector. However, no significant effects on the luciferase activity were observed due to the mutations of matching sites in the 3′-UTR of SPHK1, indicating that the specific interrelation between miRNA-103 and the binding site of the SPHK1 3′-UTR directly modulates the expression of the luciferase reporter. Next, the effects of miRNA-103 on SPHK1 expression was evaluated by the transfection with miRNA-103 inhibitor or mimic. The results showed that SPHK1 mRNA and protein were reversely regulated by miRNA-103 (Figure 4C,D).

### SPHK1 was essential to apoptosis triggered by miRNA-103 and inhibited AKT expression in chondrocytes

**Figure 5:**
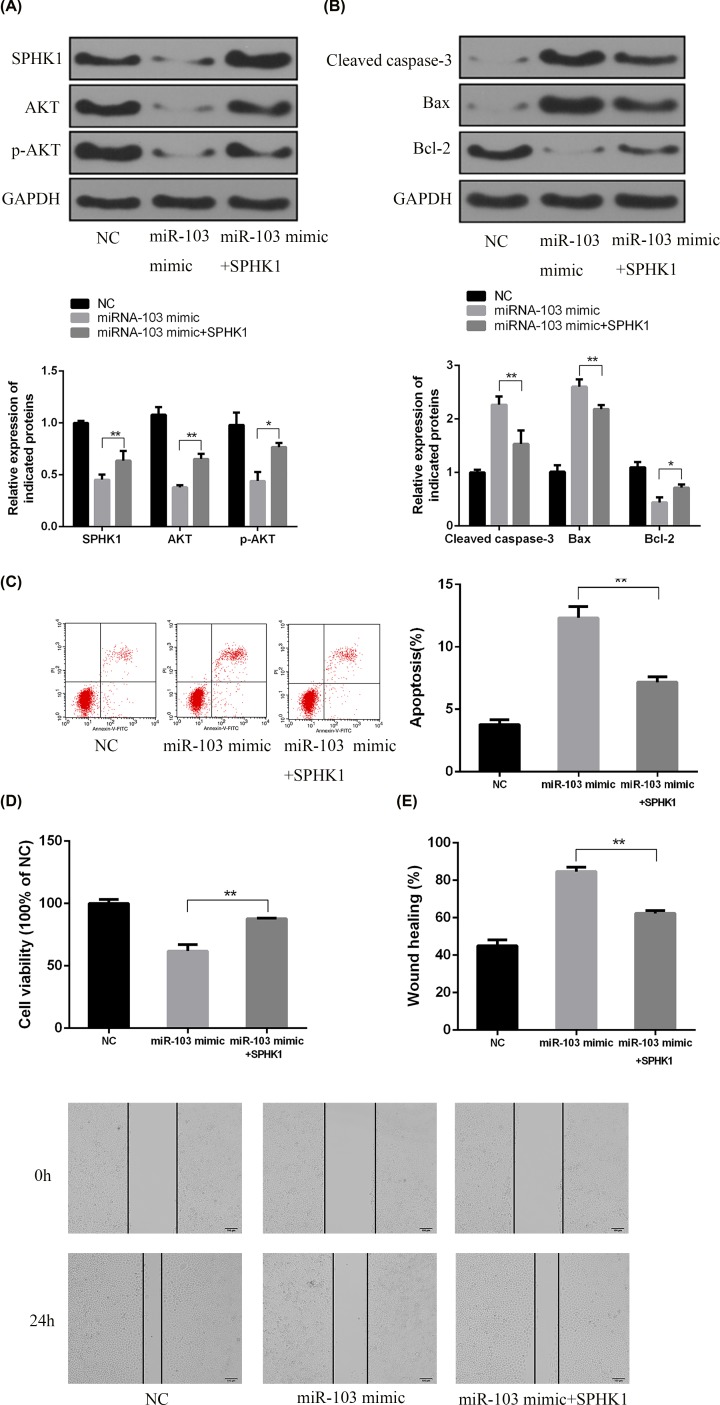
SPHK1 was essential to miRNA-103 mimic-mediated AKT inhibition and apoptosis (**A,B**) The chondrocytes were treated with miRNA-103 mimic or SPHK1 overexpression vector and indicated protein expressions were measured by Western blotting. (**C**) Percentage of apoptotic cells was measured by flow cytometry after treatment with miRNA-103 mimic and/or SPHK1 overexpression. (**D**) Cell proliferation was measured by CCK-8 assay. (**E**) Percentage of wound closure was measured by scratch wound assay. **, *P*<0.01; *, *P*<0.05.

The modulation of the PI3K/AKT pathway by miRNA-103 was determined by Western blotting. It was demonstrated that the expressions of total AKT and p-AKT were significantly reduced in chondrocytes transfected with miRNA-103 mimic. Furthermore, SPHK1 overexpression led to the up-regulated expression of p-AKT and AKT (Figure 5A).

To further confirm the roles of SPHK1 in miRNA-103-induced apoptosis, primary chondrocytes were transfected with the miRNA-103 mimic and excessively expressing SPHK1. As showed in Figure 5B, overexpression of SPHK1 inhibited the generation of cleaved caspase-3 and triggering a decline in cell death (Figure 5C). Moreover, SPHK1 up-regulation reversed the proliferation suppression induced by the miRNA-103 mimic (Figure 5D) and enhanced wound closure in comparison with cells only treated with the miRNA-103 mimic (Figure 5E).

**Figure 6:**
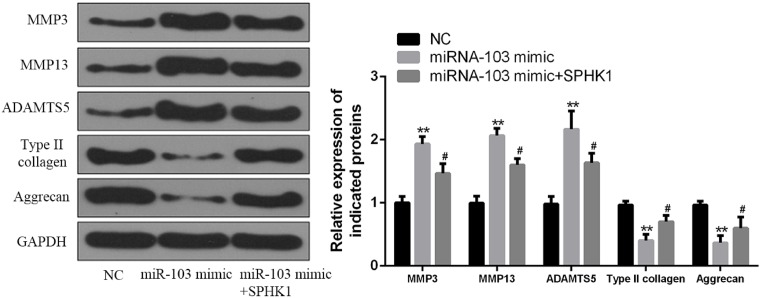
miRNA-103 modulated the expression of matrix-degrading enzymes and cartilage matrix molecules by targeting SPHK1 in chondrocytes The cells were treated as indicated, the protein level of MMP3, MMP13, ADAMTS5, type II collagen, and aggrecan were measured by Western blotting. ^#^, *P*<0.05 vs miR-103 mimic and **, *P*<0.01 vs NC.

Finally, we evaluated the effects of miR-103 on the expression of matrix-degrading enzymes and cartilage ECM molecules. As shown in Figure 6, miR-103 overexpression in chondrocytes led to increased protein levels of matrix-degrading enzymes, including MMP3, MMP13, and ADAMTS5. Consistently, miR-103 overexpression induced a significant reduction in type II collagen, and Aggrecan levels. Most importantly, SPHK1 overexpression caused a marked reduction in matrix-degrading enzymes and a marked increase in cartilage ECM molecules in miR-103-induced chondrocytes, suggesting miRNA-103 might regulate chondrosarcoma activities by down-regulation of SPHK1, at least, part through SPHK1.

## Discussion

Our present study mainly presented that miRNA-103 expression was significantly up-regulated in OA cartilage. MiRNA-103 promoted apoptosis but suppressed cell proliferation and migration in rat primary chondrocytes. In addition, SPHK1 was identified to be a direct target of miRNA-103 in rat primary chondrocytes. MiRNA-103 contributes to chondrocyte apoptosis, promoting OA progression by targeting PI3K/AKT pathway through SPHK1 activity. Silencing miRNA-103 expression may attenuate OA progression. Our present investigation may provide a therapeutic strategy for the OA treatment.

OA usually occurs in the senior age people with a joint pain and disability disease progressively developed due to damage to the articular cartilage and subchondral bone [21]. Recently, the genetic alteration from chromatin structure change, from transcription level and non-coding miRNAs regulation was associated and orchestrated the chondrogenesis and osteogenesis formation [7]. By using articular cartilage from OA patients underwent knee replacement surgery, Iliopoulos et al. [22] found out 16 differentially expressed miRNAs in osteoarthritic in contrast with normal cartilage, among which 9 miRNAs were up-regulated (i.e., miR-22 and miR-103) and 7 miRNAs down-regulated (i.e. miR29a, miR-140, and miR-25). Among these miRNAs, miR-140-ADAMTS-5 were involved in cartilage homeostasis and miR-29a-leptin were implicated in the metabolic pathways. Recently, Vonk et al. [23]reported that type II collagen and proteoglycans were increased after overexpression of hsa-miR-148a in osteoarthritic chondrocytes through inhibition of MMP-13 and ADAMTS-5 gene expression. Prior study addressed that the members of the miR-34 family are direct transcriptional targets of p53 while the ectopic expression of miR-34 induces apoptosis, cell cycle arrest, senescence, and other biological behavior [24]. In the present study, we observed that the expression of miRNA-103 was significantly increased in primary chondrocytes from OA patients compared with healthy chondrocytes, which indicates involvement of miRNA-103 in the pathogenesis of OA. To determine the function of miRNA-103 in OA, we used antisense oligonucleotides to manipulate the expression of miRNA-103. The results showed that these oligonucleotides were efficiently transfected into human chondrocyte *in vitro* and significantly increased or decreased miRNA-103 expression levels, facilitating the study of miRNA-103 function.

Mounting evidence shows that chondrocyte apoptosis plays a crucial role in the mechanisms of degeneration and degradation of articular cartilage in OA [3]. Thus, the mechanism of apoptosis offers potentially useful therapeutic targets for the management of this chronic disease [3]. In the present study, results from the flow cytometric analysis and CKK-8 assay revealed that transfection of a synthetic miRNA-103 mimic *in vitro* significantly promoted apoptosis and inhibited the cell proliferation and migration in primary rat chondrocytes, whereas the down-regulation of miRNA-103 led to significant suppression of apoptosis and enhanced cell viability.

However, only the SPHK2 isoenzyme induces apoptosis in cells [25]. In the patients with rheumatoid arthritis, synovial fluid sphingosine-1-phosphate levels were found elevated than that in OA [26]. On the contrary, the blockage of SPHK activity can dramatically ameliorate the disease of rheumatoid arthritis [27]. ABC294640, a compound that selectively inhibits sphingosine kinase-2, a key enzyme in the sphingolipid pathway, can attenuate the knee joint histological damage and pain associated with MIA-induced OA in rats [28]. However, the underlying mechanism of how SPHK1 attenuated apoptosis is unknown. To explore the underlying mechanism of the miRNA-103 involved in the OA, by bioinformatic tools, we identified the putative SPHK1 a direct target of miRNA-103 in rat primary chondrocytes. Further, we found out that SPHK1 was directly suppressed by miR-103 to block the cell proliferation. Even there is no report regarding the association between ABC294640 and miR-103, we speculated that miR-103 possibly can modulate SPHK1 and SPHK2, this will need the further examination.

PI3K and AKT can regulate the activation of the NF-κB pathway through the phosphorylation of IκBα [29]. Inhibition of the PI3K/AKT pathway and NF-κB has been considered as an option for the treatment of OA [30]. Pharmacological agents like non-steroidal anti-inflammatory drugs (NSAIDs) which inhibit the activation of NF-κB could control OA symptoms [31]. *In vitro*, the PI3K-specific inhibitor Ly294002 inhibited MMP production in chondrocytes [32]. In addition, the inhibition of AKT and NF-κB by curcumin reversed IL-1β-induced MMP secretion, COX-2 expression, and the down-regulation of type II collagen in chondrocytes. Furthermore, the inhibition of the PI3K/AKT/NF-κB pathway was able to attenuate cartilage degeneration in destabilizing the medial meniscus (DMM)-induced OA mice *in vivo* [33]. In our present study, we proved that overexpression of miR-103 suppressed the apoptosis, but increased the cell proliferation through PI3K/AKT signaling pathway by down regulation of SPHK1. Next step, we will test the inflammation index and clarify the functions of miR-103 with SPHK1, PI3K/AKT, and inflammatory events.

In summary, OA is a progressive degenerative disease characterized by cartilage degradation and chondrocyte apoptosis, the underlying mechanism may involve aberrant gene expression and the associated responding biological events. In our study, we connected miR-103 with the apoptosis, cell proliferation and through SPHK1 and AKT signaling pathway together. Our finding indicated that up-regulation of miRA-103 induced cell apoptosis and suppressed the cell proliferation through down-regulation of p-AKT pathway via blocking SPHK1 expression. The present study may provide the basis for the future therapeutic target to suppress the OA progression. Of course, our study has limitations due to the complexity of OA. We believe the mechanism of OA progression is more completed. Multifactorial molecular and signaling pathway involved in the progress. The sample amounts and statistical analysis is also our study’s limitations.

## Abbreviations

AKT: protein kinase B
CCK-8: cell counting kit-8
DMEM: Dulbecco’s modified Eagle’s medium
ECM: extracellular matrix
OA: osteoarthritis
PI: propidium iodide
PI3K: phosphatidylinsitol-3-kinase
UTR: untranslated region
