# Expression of Concern: miRNA-103 promotes chondrocyte apoptosis by downregulation of Sphingosine kinase-1 and ameliorates PI3K/AKT pathway in osteoarthritis

**DOI:** 10.1042/BSR-2019-1255_EOC

**Published:** 2024-10-07

**Keywords:** Apoptosis, miRNA-103, Osteoarthritis

The Editorial Office has been made aware of potential issues surrounding the scientific validity of this paper “miRNA-103 promotes chondrocyte apoptosis by down-regulation of Sphingosine kinase-1 and ameliorates PI3K/AKT pathway in osteoarthritis” DOI: 10.1042/BSR20191255, hence has issued an expression of concern to notify readers whilst the Editorial Office investigates. It has been noted that the Western blots appear to have minimal background details and share similarities with other Western blots present in papers by different authors. Additionally, the ethical approval statements in the Materials and Methods do not specify the location where the approval was granted. The authors were contacted regarding the concerns, however so far the Editorial Office has not received a response.

